# Through-container, extremely low concentration detection of multiple chemical markers of counterfeit alcohol using a handheld SORS device

**DOI:** 10.1038/s41598-017-12263-0

**Published:** 2017-09-21

**Authors:** David I. Ellis, Rebecca Eccles, Yun Xu, Julia Griffen, Howbeer Muhamadali, Pavel Matousek, Ian Goodall, Royston Goodacre

**Affiliations:** 1 Manchester Institute of Biotechnology, School of Chemistry, Manchester, M1 7DN UK; 2 Scotch Whisky Research Institute, Research Avenue North, Riccarton, Edinburgh, EH14 4AP UK; 3 Cobalt Light Systems Limited, Milton Park, Abingdon, OX14 4SD UK; 4 Central Laser Facility, Research Complex at Harwell, STFC Rutherford Appleton Laboratory, Harwell Oxford, OX11 0QX UK

## Abstract

Major food adulteration incidents occur with alarming frequency and are episodic, with the latest incident, involving the adulteration of meat from 21 producers in Brazil supplied to 60 other countries, reinforcing this view. Food fraud and counterfeiting involves all types of foods, feed, beverages, and packaging, with the potential for serious health, as well as significant economic and social impacts. In the spirit drinks sector, counterfeiters often ‘recycle’ used genuine packaging, or employ good quality simulants. To prove that suspect products are non-authentic ideally requires accurate, sensitive, analysis of the complex chemical composition while still in its packaging. This has yet to be achieved. Here, we have developed handheld spatially offset Raman spectroscopy (SORS) for the first time in a food or beverage product, and demonstrate the potential for rapid *in situ* through-container analysis; achieving unequivocal detection of multiple chemical markers known for their use in the adulteration and counterfeiting of Scotch whisky, and other spirit drinks. We demonstrate that it is possible to detect a total of 10 denaturants/additives in extremely low concentrations without any contact with the sample; discriminate between and within multiple well-known Scotch whisky brands, and detect methanol concentrations well below the maximum human tolerable level.

## Introduction

Spirit drinks are the EU’s biggest agri-food export, with almost two-thirds of the spirits produced in the EU being exported. As a whole, these exports and the spirits drinks industry contribute to a combined positive trade balance of around €9 billion, with EU governments’ revenues of at least €23 billion in excise duties and VAT, and approximately 1 million jobs linked to the production, distribution and sale of spirit drinks^1,2^. An essential component in ensuring consumer confidence in this industry is to provide assurance that these products are authentic and have not been either adulterated or counterfeited^3^. Importantly, sales of illicit spirit drinks not only have significant economic consequences, due to loss of trade and revenues, but can also have serious social and health impacts when ‘denatured’ alcohols or methanol (see Table 1) are consumed^4,5^.

**Table 1:** To avoid excise taxes, potable ethanol is denatured with chemicals that make it unsuitable for human consumption, and it is the excise exempt status of denatured alcohol which provides an economic incentive for its use in counterfeit spirit products.

Denaturant or flavouring	Synonym(s)	Chemical Formula	Average Mass (Da.)	Structural Formula	Concentration detected (ppm)
**Methyl ethyl ketone (MEK)**	Butanone	C_4_H_8_O	72.106		190
**Iso-propyl alcohol (IPA)**	Isopropanol	C_3_H_8_O	60.095		600
**Denatonium benzoate**	Bitrex	C_28_H_34_N_2_O_3_	446.581		0.2
**Methyl isopropyl ketone (MIPK)**	3-Methylbutan-2-one	C_5_H_10_O	86.132		6
**Ethyl sec-amyl ketone (ESAK)**	5-Methyl-3-heptanon	C_8_H_16_O	128.212		4
**Methanol (MeOH)**	Carbinol Wood alcohol	CH_4_O	32.042		250 (0.025%)
***Vanillin***	4-hydroxy-3-methoxybenzaldehyde	C_8_H_8_O_3_	152.149		10
***Sucrose***	Table sugar	C_12_H_22_O_11_	342.297		100
***Limonene***	1-Methyl-4-methylethenylcyclohexene	C_10_H_16_	136.234		100
***Trans***-***anethole***	(E)-1-(4-Methoxyphenyl) propene	C_10_H_12_O	148.202		10

The six denaturants (**bold**), and the four flavourings (***italic***) used in this study along with the minimum concentrations detected via handheld SORS and TRS are presented.

Typical modes of counterfeiting include substitution of a known brand with a cheaper product; ‘stretching’ of a genuine product with cheaper locally produced alcohol; creation of a counterfeit using a cheap local alcohol, denatured alcohol, or an alternative alcohol such as methanol, with selected flavourings and colourings added to mimic the real product (Table 1)^4^. Consequently, proving that a suspect product is non-authentic often requires an analysis of its chemical composition, to demonstrate that it is inconsistent with either its claimed category, or spirit brand. In an ideal scenario, this analysis would be undertaken *in situ* whilst the product is still in its original packaging, at any geographical location throughout a supply network. To date, a wide variety of laboratory-based techniques have been employed to authenticate spirit drinks, but these may require transportation of the samples to be analysed, are destructive and provide retrospective results.

The use of these current methods in identifying counterfeit products is typically based on the premise that the counterfeit has a distinctly different analytical profile from the genuine product. Strategies for the authentication of certain spirit categories have been reported in the literature^6–8^. With standard analytical measurement employed for the authentication of spirit drinks including: measurement of alcohol strength^4^; gas-chromatographic analysis of various volatile components^9–11^; liquid-chromatographic analyses of sugars, wood components and colouring compounds^7,12^ and the measurement of stable isotope ratios in order to provide information on either botanical^13–15^ or geographical^16^ origin. Other techniques have also been applied to the discrimination and authentication of spirits, such as: capillary electrophoresis^17^, trace element concentrations^18^; nuclear magnetic resonance (NMR) spectroscopy﻿ ^19,20^ and electrospray ionization mass spectrometry^21,22^.

In addition to a range of laboratory-based methods, attention has moved to the authentication of foods and beverages using spectroscopic techniques^23–25^ that offer the potential to be employed in rapid^26^, portable applications^27–29^. Such spectroscopic techniques that have been specifically applied to the analysis of spirit beverages include: UV-Vis spectroscopy^30–32^, mid-infrared spectroscopy^33^, near infrared^34^ and Raman^34,35^. However, only one of these latter studies, by Nordon and co-workers^34^ was non-invasive and through-container, using Raman and NIR to measure ethanol concentration through clear glass bottles, with the authors stating that strong absorption/fluorescence occurred with coloured glass bottles.

Here, for the first time in a food or beverage product, we use handheld spatially offset Raman spectroscopy (SORS)^36^, as well as the closely related technique, transmission Raman spectroscopy (TRS)^37^. These approaches enable the isolation of chemically-rich spectral data from subsurfaces, substructures, layers and through other types of barriers, not as readily, deeply, or widely accessible via conventional Raman spectroscopy^38^. Two situations were considered: a) the ability to discriminate between and within spirit brands on the basis of their Raman spectra, and more importantly here, b) the potential of rapid, through-container and unequivocal detection of multiple illicit chemical markers that signify the presence of counterfeit spirit with this new technology. This was undertaken using a range of key chemical markers, specifically targeted as they are commonly used to denature or flavour alcohol, and are regularly found in counterfeit spirits around the globe.

## Materials and Methods

**Figure 1:**
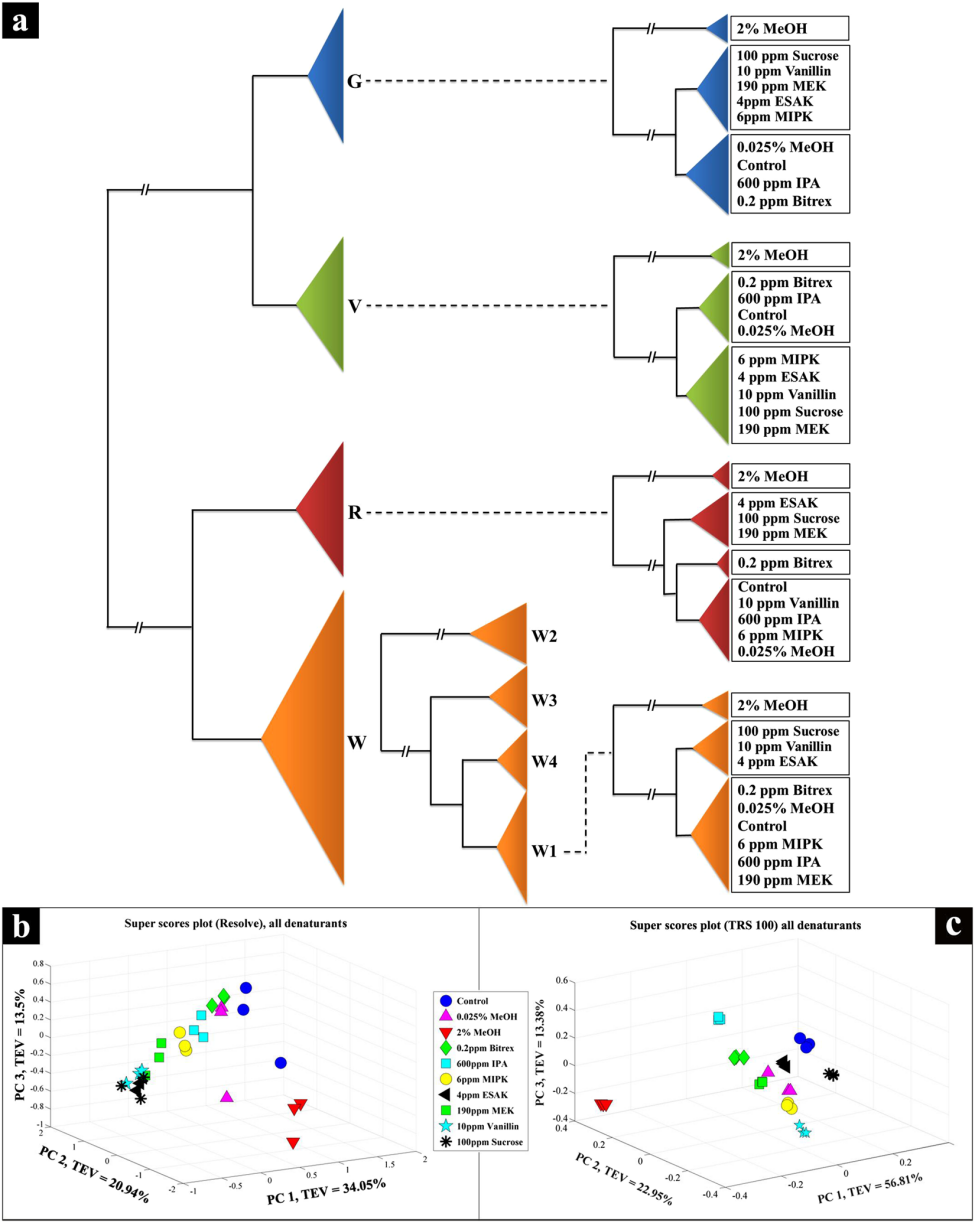
(**a**) Abridged dendrogram obtained by hierarchical cluster analysis of the PC-DFA scores of all the Raman spectral data collected using SORS; encoding used: gin (G), vodka (V) rum (R), 4 brands of whisky (W1-W4). MB-PCA super scores plots of the Raman spectral data collected using, (**b**) SORS with a Resolve instrument and, (**c**) TRS with a TRS100 instrument. Different symbols represent the different additives, and the abbreviations are shown in Table 1.

A total of 144 samples were provided by the Scotch Whisky Research Institute (SWRI). These were analysed blinded and represent widely consumed and popular brand profiles: a) 61 different production rotations of a genuine Scotch whisky (W1); b) 10 different production rotations of a second genuine Scotch whisky (W2); c) 10 different rotations of a third genuine Scotch whisky (W3); d) 10 different rotations of an Irish whiskey (W4); and e) 10 different rotations of a vodka (V) (Fig. 1). All of these genuine products were supplied by their producers to the SWRI. Samples were aliquoted directly into identical, 2.3 mL clear glass threaded vials and closed with caps on-site at SWRI in Edinburgh for analysis in Oxford and Manchester, UK. Also included were multiple simulated counterfeit products (*n* = 40), where a number of compounds detailed in Table 1 were added to vodka, whisky, gin and rum. These compounds include: i) flavourings commonly found in counterfeit whisky samples such as vanillin, sucrose, limonene and trans-anethole; ii) methanol due to the health implications of its consumption; and iii) denaturants that were commonly used in Europe prior to Commission Implementing Regulation (EU) 2016/1867. The vodka, whisky, gin and rum used as diluents were created by homogenising a bulk volume of each spirit category, in a beaker on a magnetic stirrer plate for 1 h.

The initial recipe for the European method of completely denatured alcohol prior to Commission Implementing Regulation (EU) 2016/1867 was 3 L of Isopropyl Alcohol (IPA), 3 L of Methyl Ethyl Ketone (MEK) and 1 g of Denatonium Benzoate per 100 L of pure ethyl alcohol (Commission Implementing Regulation (EU) 162/2013). At the time, there was also a popular German formulation that stated per 100 L of alcohol a 1 L ketone mixture should be added along with 1 g of denatonium benzoate, the ketone mixture consisting of 95-96% by weight methyl ethyl ketone, 2.5–3% by weight methyl isopropyl ketone and 1.5–2% by weight ethyl sec-amyl ketone (Commission Implementing Regulation (EU) 162/2013). This information was used to prepare an appropriate sample test set. After cost concerns were raised during the regulatory consultation period, the released final formulation in Commission Implementing Regulation (EU) 2016/1867 reduced the quantity of IPA and MEK from 3 L to 1 L per 100 L of pure ethyl alcohol.

The simulated counterfeit spirits were created to represent useful and real-world levels of detection in the case of flavourings and methanol; and to establish the possibility of identification of denatured (and therefore tax free) alcohol in a range of different matrices (multiple brands as well as different types of spirit drinks). The dilutions represent 1/20^th^ of the concentration of the denatured alcohol at 40%. For example, denatonium benzoate is a denaturant used at 1 g/100 L of pure ethyl alcohol. If this is reduced to 40% alcohol, the strength typically seen in spirit drinks, the concentration would be 4 mg/L. Due to the bitter taste of denatonium benzoate it is likely that a counterfeiter would attempt to remove the denaturant or dilute it further with genuine spirit. For these experiments the further dilution or attempted removal is represented by diluting the samples to 1/20^th^ of the concentration seen in 40% spirit giving 0.2 mg/L of denatonium benzoate in the test samples. The full list of chemicals used for the simulated counterfeit spirits, their Chemical Abstracts Service (CAS) number, concentrations and suppliers can be seen in Table 2 below. All of the denaturants and flavourings were prepared individually in vodka (a simple spirit) and whisky (a complex spirit), trans-anethole and limonene were not prepared in rum or gin as they may be permitted in genuine products of these categories, ‘blank’ samples were also provided.

**Table 2:** Chemicals used in creating the simulated counterfeit samples analysed in this study.

Chemical name	CAS number	Concentration	Supplier
Ethanol	64-17-5	various	Rathburn chemicals
Ultra-High Quality Water produced by ELGA PURELAB Option-Q7/15 benchtop generator	7732-18-5		
0.025% MeOH	67-56-1	0.025% v/v	Rathburn chemicals
MeOH 2% (maximum tolerable human intake in 40% spirit)	67-56-1	2% v/v	Rathburn chemicals
Denatonium benzoate	3734-33-6	0.2 mg/L	Sigma Aldrich
Isopropyl alcohol (IPA)	67-63-0	600 mg/L	Sigma Aldrich
Methyl isopropyl ketone (MIPK)	563-80-4	6 mg/L	Tokyo Chemical Industry
Ethyl sec-amyl ketone	541-85-5	4 mg/L	Tokyo Chemical Industry
Methyl ethyl ketone, MEK	78-93-3	190 mg/L	Sigma Aldrich
Vanillin	121-33-5	10 mg/L	Sigma Aldrich
Sucrose	57-50-1	100 mg/L	Sigma Aldrich
Limonene	5989-27-5	10 mg/L	Sigma Aldrich
Trans-anethole	4180-23-8	10 mg/L	Sigma Aldrich

The manner of sample preparation for the simulant samples used parent standards in 100% of pure ethyl alcohol (Rathburn Chemicals), where the chemical was accurately weighed into a volumetric flask and made up to volume with the ethanol. Working standards in 40% ethanol/ ultra high quality water (UHQ) were then created; these were prepared so that the same volume of working standard was diluted into each diluent for all chemicals used. In order to avoid dilution effects the same volume of ‘blank’ 40% ethanol/UHQ was then added to the blank samples.

Spectroscopic analysis was undertaken using a Resolve (Cobalt Light Systems, UK) handheld SORS through-barrier identification system which operates using a 450 mW, 830 nm laser at typically 3 cm^−1^ resolution. Principally designed for the identification of hazardous materials, explosives, and raw pharmaceutical compounds^39^, this instrument has three modes of analysis: through-barrier, surface scan, and vial mode. Here, this portable instrument was used purely as a spectrometer as a means of obtaining spectra for off-line analysis. In addition, and for comparative analysis, the same samples were also analysed using a benchtop TRS100 (Cobalt Light Systems, UK) transmission Raman system which operates using a 650 mW, 830 nm laser at 8 cm^−1^ resolution. For TRS the Raman signal is collected on the opposite side of irradiation and an automated tray system moves samples (in vials) into the measurement position. For handheld SORS (Resolve) Raman spectra were collected using the vial holder adapter which encloses vials in a chamber preventing stray light interference. All 144 (randomised) spirit samples analysed by both methods were undertaken in triplicate with a total of 864 Raman spectra collected for subsequent data pre-processing and multivariate statistical analysis. Individual collection times were 30 s for handheld SORS (Resolve) and 7 s for TRS (TRS100) spectra. All the measurements presented here (SORS and TRS) were undertaken through the glass vials.

In addition to the 144 samples provided in closed glass threaded vials by SWRI, analysis of an additional subset of samples was undertaken by handheld SORS (Resolve) on shop bought bottles of spirit drinks. This subset was undertaken primarily to test if this approach had the capability to be transposable and acquire practicable Raman spectra through commercially available clear and coloured glass bottles of spirits. In order to achieve this, three 50 mL bottles of branded spirit drinks were purchased from national retail outlets, vodka (37.5% alcohol by volume (ABV)) in clear (flint) glass, Scotch whisky (46% ABV) in green glass, and gin (47% ABV) in brown glass.

**Figure 2:**
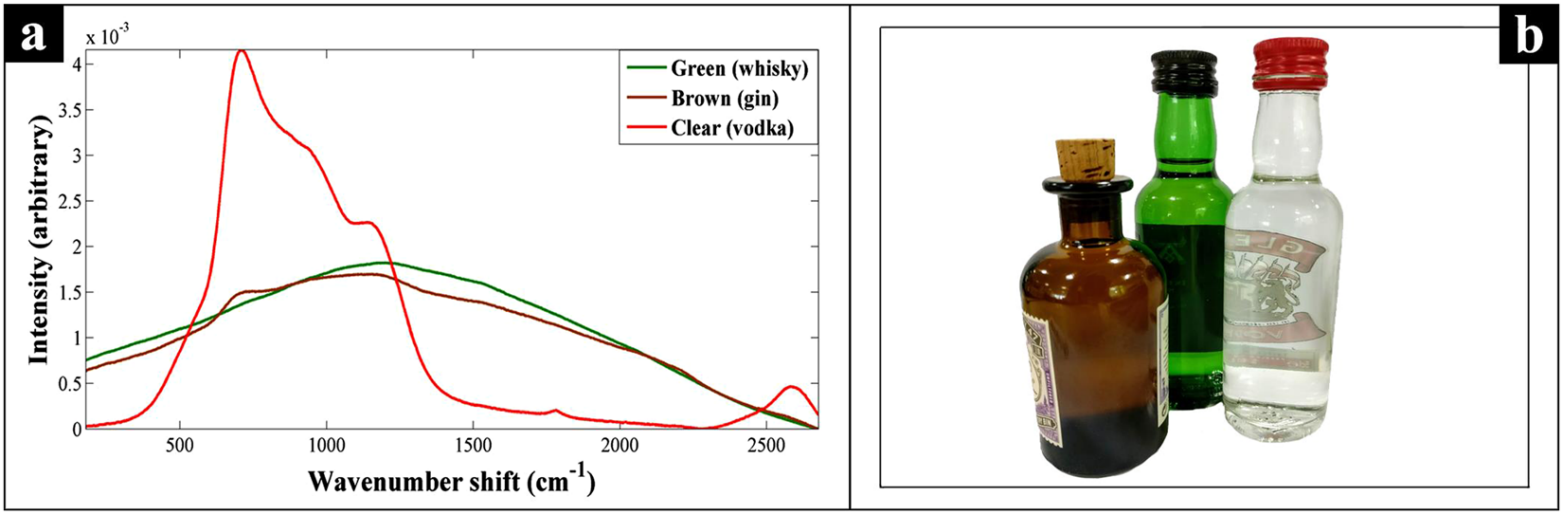
The zero measurement SORS spectra (**a**) of bottle glass from three commercial spirits drinks (**b**) vodka in clear glass bottle, gin in brown glass bottle, and Scotch whisky in green glass bottle. Resolve is a handheld SORS (through-barrier) device which takes two measurements, a zero and an offset. The difference between these is that the laser physically moves. The *zero* measurement can be thought of as traditional backscatter mode, with the Raman signal acquired being biased to the surface. So in the case of the glass bottles that we see here (**b**), it is generally just the fluorescence of the glass that is observed.

Spectra were collected with the handheld Resolve device using the through-barrier measurement setting and the standard nose cone adapter. Glass bottles were shrouded with a black cloth to prevent stray light interference. Acquisition settings were set to scan for 60 accumulations in the offset position to improve signal quality. Total scan time was approximately 60–90 s. Six replicates were collected from each of the three spirits (Scotch whisky, gin, vodka) and glass types (green, brown, and clear), the first set of replicates were collected from each spirit within their unopened bottles (0% adulteration). Bottles were then opened and each individual spirit sequentially adulterated with 1%, 2%, and 3% methanol respectively. Six replicates were collected from each of these levels of methanol adulteration in addition to unopened and unadulterated spirit, with a total of 72 Raman spectra collected for subsequent data analysis. All the measurements presented for this test subset were undertaken through three different colours of commercially available glass bottles of spirit drinks (Fig. 2).

The Raman spectra were first filtered with a moving median filter with a window width of 3 bins (3 cm^−1^ for the TRS data and ~9 cm^−1^ for the SORS data) to remove unavoidable cosmic rays. The standard normal variate (SNV) method^40^ was then employed for data pre-processing to normalize the Raman spectra obtained by both devices. Principal component analysis (PCA)^41^ was applied to these pre-processed Raman spectra. The natural relationships in the samples were revealed in the PCA scores plots while the variables (i.e., wavenumber shifts) contributing to these models were illustrated in the corresponding PCA loadings plots. Multi-block principal component analysis (MB-PCA)^42^ was also applied to the Raman spectra of samples with denaturants added to highlight the differences between these samples. The “common trend” across all four different spirits (i.e., whisky, gin, vodka and rum) was provided in the super scores plots while the individual pattern of each spirit was presented in the corresponding block scores plots. Similar to PCA, the contributions of the variables were given in the loadings plots. Finally, an abridged dendrogram was constructed by performing hierarchical cluster analysis (HCA) on the principal component-discriminant function analysis (PC-DFA) scores (first 5 PCs, which typically account for > 99% total explained variance (TEV)) using Euclidean distance and average linkage (Fig. 1).

## Results and Discussion

Spirit drinks have a complex chemistry and especially those, such as Scotch whisky for example, that undergo maturation in wooden casks. The major chemical compound of importance is of course ethanol, which has also been at the forefront of many previous studies as the target of detection and quantification for multiple analytical methods. From our own analysis, it was evident that Raman spectra (SI 1.) were indeed dominated by ethanol vibrations (C-C stretch at 892, C-O stretch at 1059 and 1097, and CHx bend at 1460 cm^−1^)^43^. Other chemicals which can be present within whiskies include phenolic compounds such as guaiacol, cresols, xylenol and eugenol, aldehydes, a range of esters, the so-called whisky lactones, in addition to many others^44–47^. It is this complexity and the resulting variation in flavours and aromas, which can be said to contribute to the global popularity of Scotch whisky. Yet it is this very same heterogeneous chemical matrix that also presents the analyst with significant challenges when attempting to detect the presence of extremely low concentrations of other chemical marker compounds, and especially so through closed containers.

Here, using handheld SORS and TRS we have shown that it is possible to achieve several goals without having any contact with the sample itself. Using simple PC-DFA and HCA of the SORS spectra, as illustrated in the abridged dendrogram (Fig. 1a), we demonstrate that it is readily possible to discriminate between all four types of spirit drink under analysis and further differentiate the four blended whiskies (W1-W4), which are more economically significant and arguably more chemically complex than whiskies from single distilleries. We have also detected different levels of ethanol (40% and 43% ABV) within the same blend of whisky (see SI.2). More importantly, the MB-PCA showed that methanol was detected, not only at its maximum tolerable level^48,49^ of 2% but as low as 0.025% (Fig. 1b,c). Furthermore, we have successfully detected five other adulterants that are markers of illicit alcohol, the consumption of which is becoming increasingly common and causing multiple deaths worldwide^50–55^ and four flavouring compounds which also act as markers of counterfeit whisky (Fig. 1b,c). This range of six denaturants and four additives were all detected through glass at extremely low concentrations, four of them at or below 10 ppm for example, with the lowest, denatonium benzoate, detected at 0.2 ppm (Table 1).

The TRS and handheld SORS methods both gave very good results and compared well (SI 3., SI 4., SI 5.), with improved discrimination observed from groups of the spirit sample types using handheld SORS. This could be related to the longer spectral acquisition times of handheld SORS compared to TRS, as well as differences in detector types for these two methods. With TRS operating with a fibre-coupled spectrograph and detector, whilst handheld SORS (Resolve) operates with a miniaturised optical engine which uses a free space detector. Whilst we observed slightly improved results from handheld SORS in direct comparison to TRS on the same samples (likely due to acquisition times), it is in this regard where benchtop TRS has its own advantages. As the faster acquisition times and TRS100 tray system enables high-throughput analyses of batches of samples if so required, with ~30 vials in a single measurement session.

**Figure 3:**
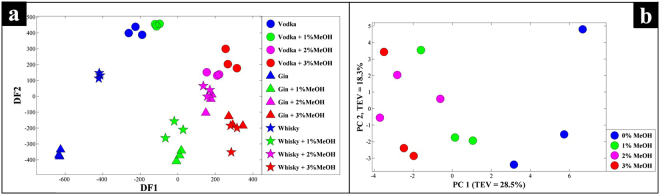
The PC-DFA scores plot (**a**), and MB-PCA super scores plot (**b**) of the Raman spectral data collected using SORS with a Resolve instrument through commercial glass bottles (Fig. 2b). Different symbols represent the different spirit types, and the colours indicate the level of methanol present in each of the samples.

Next, in order to test if these methods had the real potential to be transposable to commercially-bottled samples, the handheld SORS device was used to measure three samples of spirits drinks purchased ‘off the shelf’. It was notable that there were observable differences in these data related to the type of coloured (green/brown) or clear glass. With the darker colours of green and brown glass allowing less light, and therefore Raman signal to pass through (Fig. 2). Due to these effects the collected Raman spectra had a lower signal-to-noise ratio than those obtained through clear sample vials, thus five to nine spectra were taken from each sample and the top three with the highest signal-to-noise ratio used for analysis. The PC-DFA scores plot (Fig. 3) of Raman spectral data, collected by handheld SORS from the three different spirits through their commercial glass bottles, displayed a clear separation of the spirits according to their methanol content on the DF1 axis. It is also evident from the DF1 loadings plot (SI. 6) that the peak at 1023 cm^−1^ is a significant contributor to the separation on this axis, which can be assigned to the C-O stretch of methanol. In addition, the DF2 axis also displayed separation of the different types of spirits, especially prior to adulteration (Fig. 3a). The MB-PCA super scores plot (Fig. 3b) was in agreement with the above findings, as it also displayed clear differentiation of samples according to methanol content on the PC1 axis with a TEV of 28.5%. The individual MB-PCA spirit-blocked scores plots (SI 7.) also displayed clear separation of the samples according to their methanol content.

The differentiation of these groups of spirit drinks within their commercial glass bottles via handheld SORS is not only a result of their complex chemistry (particularly in the case of gin and Scotch whisky), but very likely a combination of spirit type, alcohol (ethanol/methanol) concentrations, and glass opacity. With the handheld SORS device correcting for this and detecting adulteration with methanol through multiple colours of commercial glass bottles in several spirit drinks, well below the maximum human tolerable level. We believe that these data demonstrate the transposable nature of our handheld SORS approach and its potential for use in commercial glass bottles of multiple spirits drinks in coloured as well as clear glass bottles.

## Conclusion

SORS is already showing considerable promise for the detection of explosives and other hazardous materials and are common-place in security screening in airports throughout Europe^56^. We believe that photonics approaches such as handheld SORS and TRS have significant and, as of yet, untapped potential for their application to food security challenges. Especially so with further technological developments, such as continued miniaturisation^57^, leaps in portable battery/energy storage and wearables^58–61^, networked with remote access sensor/actuator capabilities that would detect global trends in food security risks^62^, or simply as highly mobile and user-friendly handheld sampling devices taken out of laboratory settings and into complex logistical supply chains for rapid on-site analysis^29^. Moreover, these methods have the potential to be very useful in identifying authentic food and drink products, in an era when there is growing evidence that counterfeiting of Geographical Indications (GI)^63^ (which includes many spirit drinks) for example, is on the increase in the EU and elsewhere^64^. And sampling, a term (along with *testing*) that is easily misunderstood and at times misused outside the natural and clinical sciences, has been said to be one of the most effective ways of harvesting and developing information on the true magnitude of counterfeiting^65^. With technological sampling methods, such as the handheld through-container approaches we are forwarding here, being practicable, and, as stated elsewhere, with the expectation that they generate unbiased results with fewer assumptions than other conventional or subjective approaches^64^.

## Electronic supplementary material

Supplementary Information
