# Supplementary material for: Through-container, extremely low concentration detection of multiple chemical markers of counterfeit alcohol using a handheld SORS device
Source: Sci Rep. 2017 Sep 21;7:12082. doi: 10.1038/s41598-017-12263-0 (PMC5608898; doi:10.1038/s41598-017-12263-0)

Supplementary information

Through-container, extremely low concentration detection of multiple chemical markers of counterfeit alcohol using a handheld SORS device

Ellis, D.I.[1*], Eccles, R.[2], Xu, Y.[1], Griffen, J.[3], Muhamadali, H.[1], Matousek, P.[3,4], Goodall, I.[2], and Goodacre, R[1*]

[1]Manchester Institute of Biotechnology, School of Chemistry, Manchester, UK, M1 7DN, [2]Scotch Whisky Research Institute, Research Avenue North, Riccarton, Edinburgh, UK, EH14 4AP, [3]Cobalt Light Systems Limited, Milton Park, Abingdon, OX14 4SD, UK, [4]Central Laser Facility, Research Complex at Harwell, STFC Rutherford Appleton Laboratory, Harwell Oxford OX11 0QX, UK.

*Corresponding authors: D.Ellis@manchester.ac.uk Roy.Goodacre@manchester.ac.uk

SI 1. Raman spectra of pure spirits acquired using (a) Resolve and (b) TRS100 instruments. Each spectrum represents average of multiple spectra collected from each class.

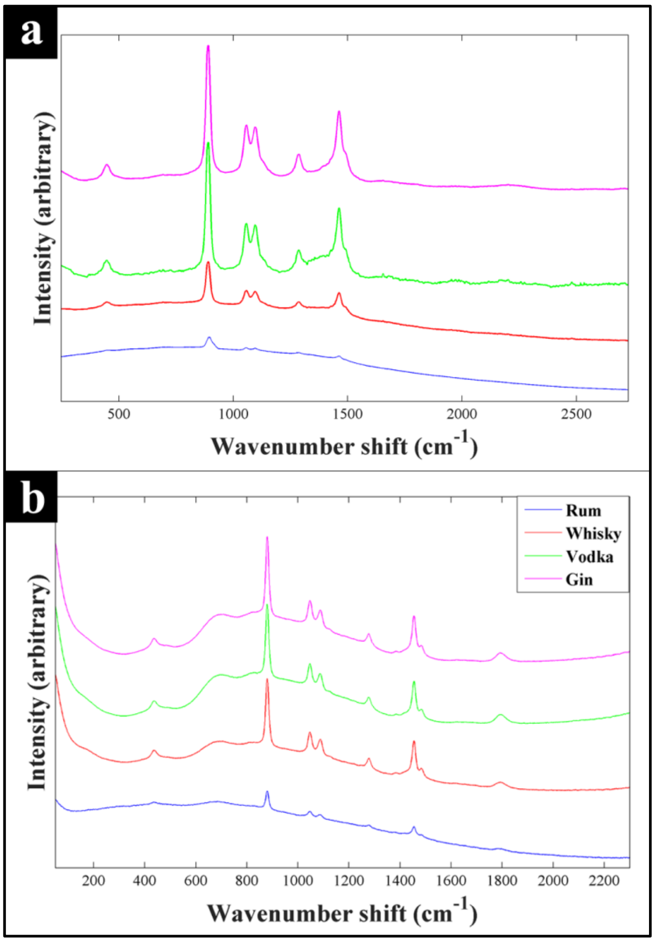

SI 2. PCA scores plots of Raman spectral data collected using (a, b) Resolve and (c, d) TRS100 instruments. Different samples are presented by different symbols and colours, while denaturant containing samples are presented as empty symbols. b) W1-14 indicates Scotch whisky samples that were 43% ethanol concentration.

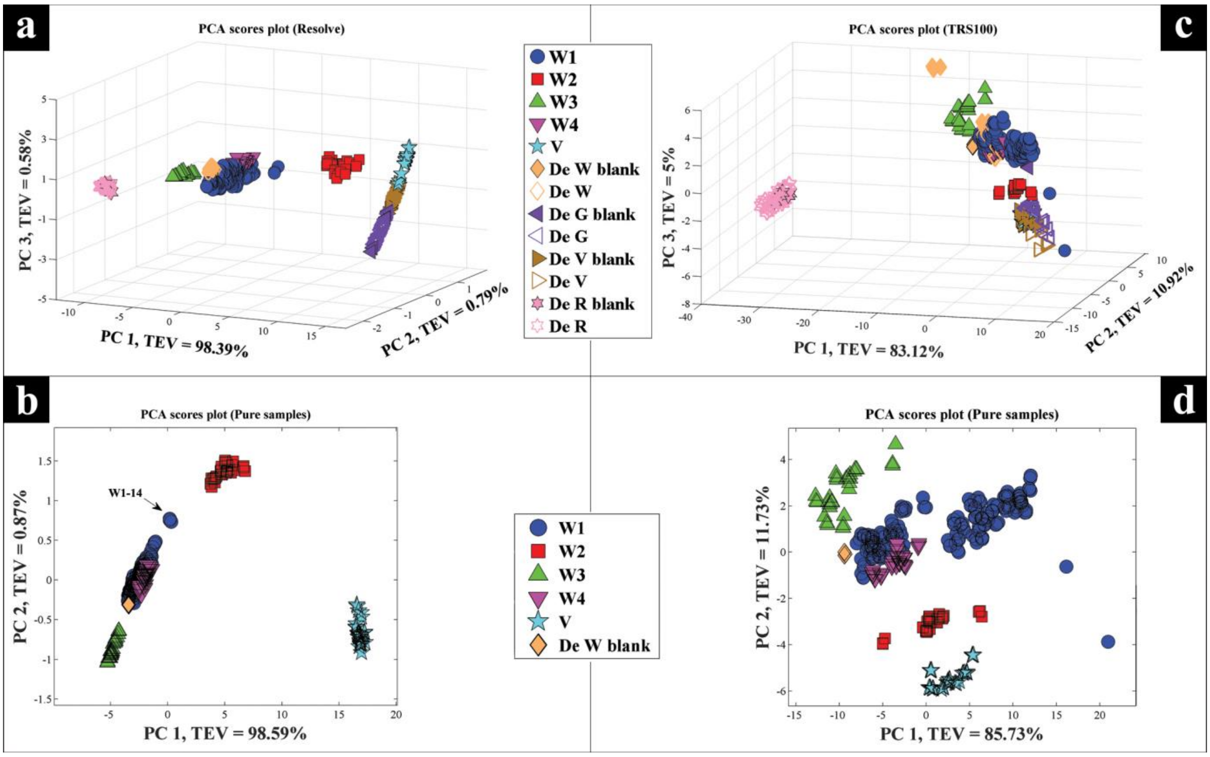

SI 3. MB-PCA scores plots of Raman spectral data collected using the Resolve instrument. Each block displays clustering of Raman spectral data in a single class (spirit), while different symbols represent samples with different additives.

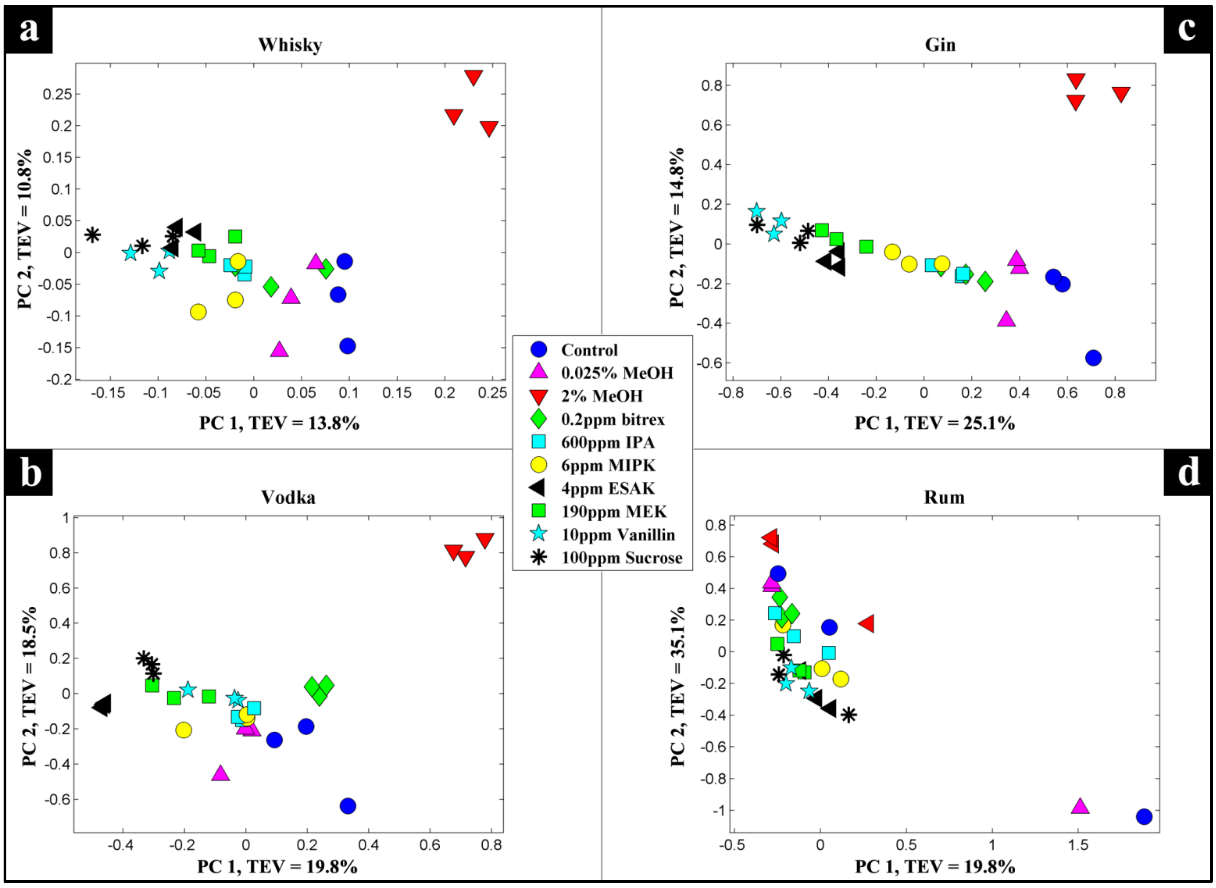

SI 4. MB-PCA scores plots of Raman spectral data collected using the TRS100 instrument. Each block displays clustering of Raman spectral data in a single class (spirit), while different symbols represent the different additives.

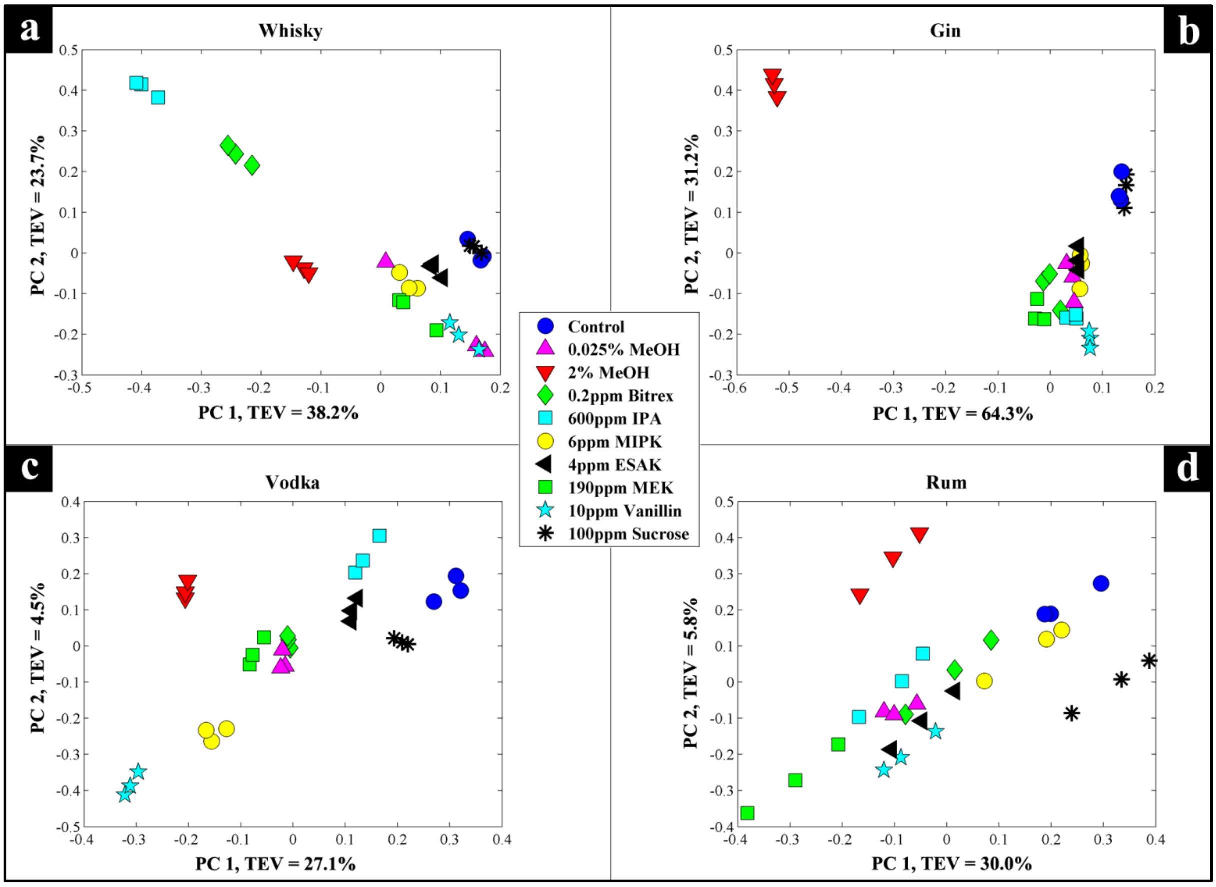

SI 5. PCA scores plots of Raman spectral data of all whisky samples collected using the a) Resolve and b) TRS instruments. Different symbols represent samples with different additives. These samples contained all four compounds vanillin, sucrose, limonene and trans-anethole which are often found as added flavourings in counterfeit whisky.

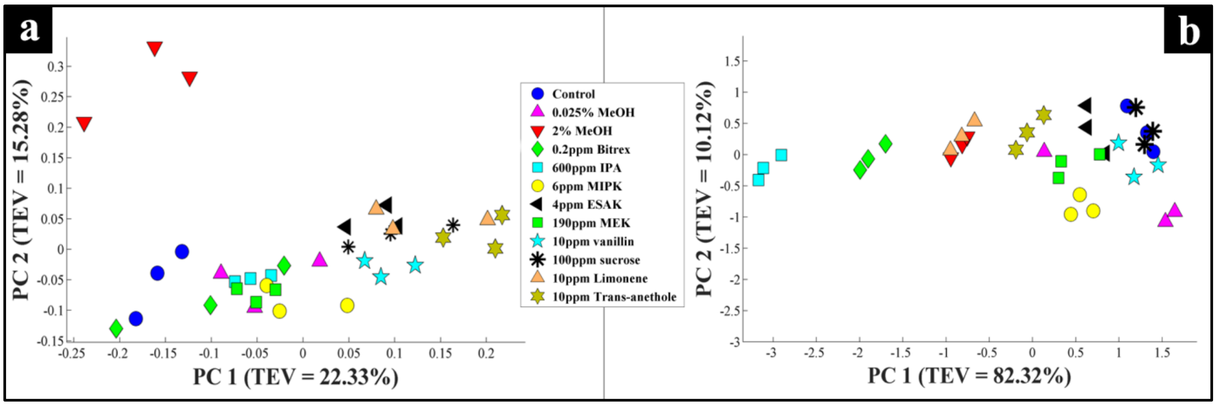

SI 6. The DF1 loadings plot from Raman spectral data collected by handheld SORS from three commercial glass bottles of various colours. The methanol peak at 1023 cm^{-1} is highlighted.

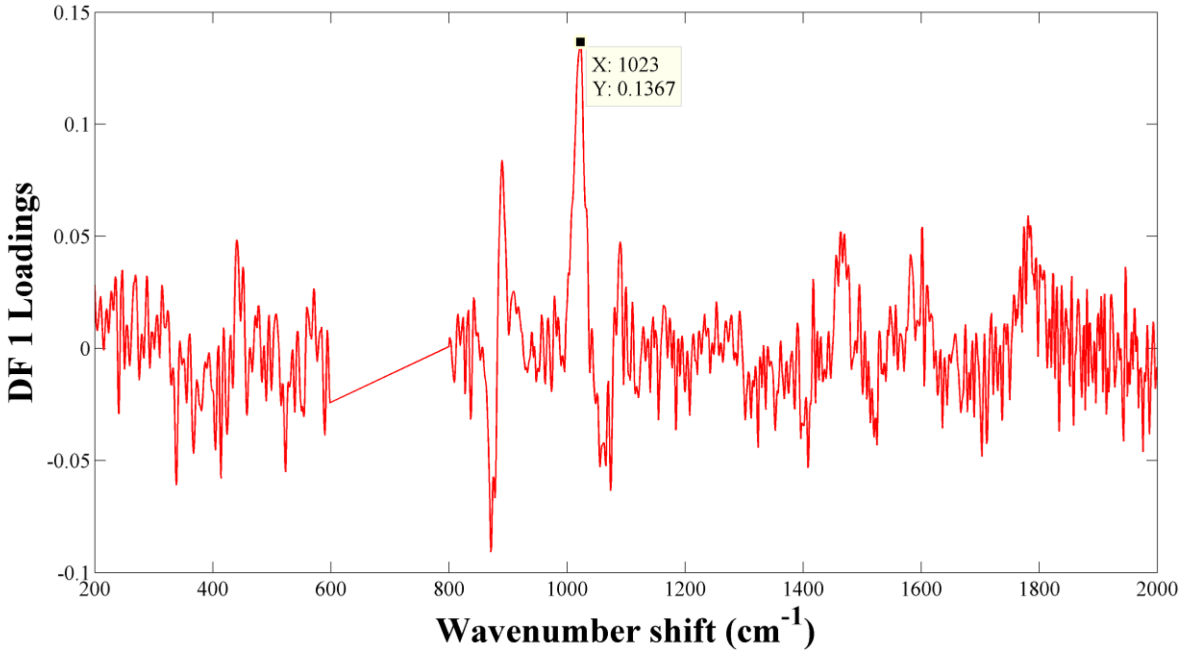

SI 7. MB-PCA scores plots of the individual spirit-blocked Raman spectral data collected from commercial glass bottles using SORS with a Resolve instrument. Different symbols represent the different spirit types, and the colours indicate the level of methanol present in each of the samples. Vodka - clear glass bottle, Whisky - green glass bottle, Gin - brown glass bottle.

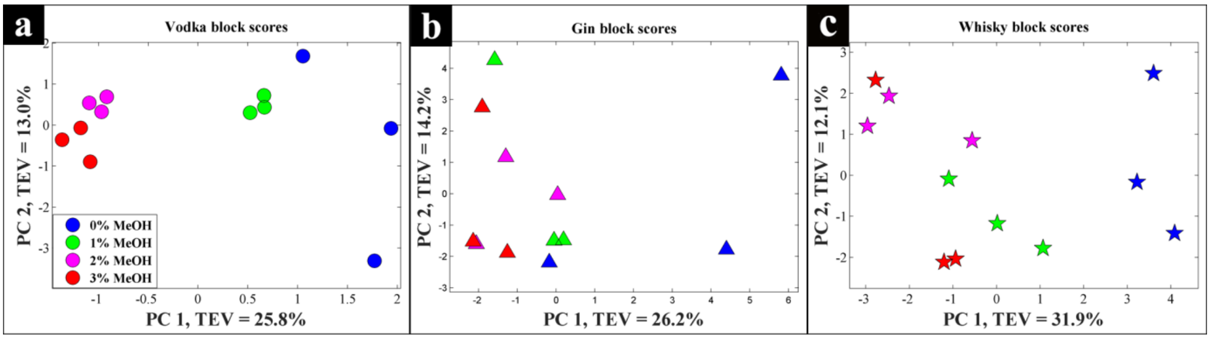

Supplement: Supplementary file 1 — Supplementary Information [file 41598_2017_12263_MOESM1_ESM.pdf]
